# Genome-wide DNA methylome variation in two genetically distinct chicken lines using MethylC-seq

**DOI:** 10.1186/s12864-015-2098-8

**Published:** 2015-10-23

**Authors:** Jinxiu Li, Rujiao Li, Ying Wang, Xiaoxiang Hu, Yiqiang Zhao, Li Li, Chungang Feng, Xiaorong Gu, Fang Liang, Susan J. Lamont, Songnian Hu, Huaijun Zhou, Ning Li

**Affiliations:** The State Key Laboratory for Agro-biotechnology, China Agricultural University, Beijing, 100193 China; Core Genomic Facility, Beijing Institute of Genomics, Chinese Academy of Sciences, Beijing, 100101 China; Department of Animal Science, University of California, Davis, CA 95616 USA; Department of Animal Science, Iowa State University, Ames, IA 50011 USA; CAS Key Laboratory of Genome Sciences and Information, Beijing Institute of Genomics, Chinese Academy of Sciences, Beijing, 100101 China; Department of Poultry Science, Texas A&M University, College Station, TX 77845 USA; National Engineering Laboratory for Animal Breeding, China Agricultural University, Beijing, 100193 China; College of Animal Science and Technology, Yunnan Agricultural University, Kunming, Yunnan China

**Keywords:** Epigenetics, DNA methylation, MethylC-seq, Immunity, Chickens

## Abstract

**Background:**

DNA cytosine methylation is an important epigenetic modification that has significant effects on a variety of biological processes in animals. Avian species hold a crucial position in evolutionary history. In this study, we used whole-genome bisulfite sequencing (MethylC-seq) to generate single base methylation profiles of lungs in two genetically distinct and highly inbred chicken lines (Fayoumi and Leghorn) that differ in genetic resistance to multiple pathogens, and we explored the potential regulatory role of DNA methylation associated with immune response differences between the two chicken lines.

**Methods:**

The MethylC-seq was used to generate single base DNA methylation profiles of Fayoumi and Leghorn birds. In addition, transcriptome profiling using RNA–seq from the same chickens and tissues were obtained to interrogate how DNA methylation regulates gene transcription on a genome-wide scale.

**Results:**

The general DNA methylation pattern across different regions of genes was conserved compared to other species except for hyper-methylation of repeat elements, which was not observed in chicken. The methylation level of miRNA and pseudogene promoters was high, which indicates that silencing of these genes may be partially due to promoter hyper-methylation. Interestingly, the promoter regions of more recently evolved genes tended to be more highly methylated, whereas the gene body regions of evolutionarily conserved genes were more highly methylated than those of more recently evolved genes. Immune-related GO (Gene Ontology) terms were significantly enriched from genes within the differentially methylated regions (DMR) between Fayoumi and Leghorn, which implicates DNA methylation as one of the regulatory mechanisms modulating immune response differences between these lines.

**Conclusions:**

This study establishes a single-base resolution DNA methylation profile of chicken lung and suggests a regulatory role of DNA methylation in controlling gene expression and maintaining genome transcription stability. Furthermore, profiling the DNA methylomes of two genetic lines that differ in disease resistance provides a unique opportunity to investigate the potential role of DNA methylation in host disease resistance. Our study provides a foundation for future studies on epigenetic modulation of host immune response to pathogens in chickens.

**Electronic supplementary material:**

The online version of this article (doi:10.1186/s12864-015-2098-8) contains supplementary material, which is available to authorized users.

## Background

DNA methylation is a central epigenetic modification that occurs in most eukaryotic organisms and plays a crucial role in transcriptional regulation. This epigenetic mark is involved in many cellular processes, including embryogenesis, transposon silencing, genomic imprinting, X chromosome inactivation, and tumorigenesis [1–3].

Of the many approaches to profile genome-wide DNA methylation patterns, MethylC-seq is considered the current “gold standard” [4, 5]. Compared with other methods, this approach achieves higher resolution and more precise methylation levels at the single-base resolution. Due to the significant role of DNA methylation on biological processes, recent studies have focused on genome-wide DNA methylation pattern in different eukaryotic genomes. To date, single-base resolution DNA cytosine methylome maps for Arabidopsis, human, silkworm, and chicken have been generated by MethylC-seq [3, 6–9].

The chicken (*Gallus gallus*), as a representative of extant avian species, is a model organism [10] for studying embryology, immunology, behavior, and reproduction [11, 12]. Furthermore, avian species are reservoirs of many zoonotic pathogens and, therefore, studies of their genomes provide new insights into how the host responds to pathogens of biomedical importance [11, 13, 14]. Although the chicken genome has been sequenced, one-dimensional sequencing information can only address a fraction of the relevant biological questions [15]. The chicken genome has an active DNA methylation system [16, 17]; therefore, it is of interest to decipher the methylation-determined epigenetic landscape of the chicken. Whole-genome DNA methylation profiling of the chicken was previously conducted using MeDIP and Methyl-MAPS [18–20]. Because of the technical limitations inherent in these techniques, such as low resolution and/or complexity, only a reduced landscape of the DNA methylome was generated. Recently, single-base resolution DNA methylome sequencing of chicken sperm cells was accomplished [9]. Because of the unique nature of high methylation levels in sperm cells, however, single-base high-resolution methylome information on other tissues in the chicken is needed to enhance our understanding of genome-wide profiles of DNA methylation in this species.

Disease resistance has been one of most challenging traits to enhance in the poultry breeding industry. During domestication and genetic selection, different chicken breeds have displayed a variety of resistance levels to pathogens. For example, the Fayoumi chicken, which originated in Egypt, has been demonstrated to resist viral, bacterial and parasitic infections including Marek’s disease virus, avian influenza virus (AIV), *Salmonella enteritidis* and *Eimeria* coccidiosis [21–25]. The Leghorn line used in the current study, which was derived from egg-laying stock in the U.S., is relatively susceptible to pathogen infection compared to the Fayoumi [26].

Previous studies have suggested that abnormal DNA methylation contributes to cancer and infectious disease [27, 28]. To explore potential regulatory roles of DNA methylation related to disease resistance, we generated whole genome single-base DNA methylation profiles of two highly inbred Fayoumi and Leghorn lines (inbreeding coefficient >99.99 %). These two chicken lines have been studied for many facets of disease resistance and immune response [26, 29–31]. Their high inbreeding level minimizes within-line genetic variation and their distinct pathogen responses between lines provide an opportunity to identify DNA methylation differences between disease resistant and susceptible chicken lines and to explore the potential biological role of DNA methylation on immune response. In addition, transcription profiling of the same tissues used in the DNA methylation analysis was performed to interrogate the relationship between DNA methylation and transcriptional regulation on a genome-wide scale.

## Results

### Single-base resolution DNA methylome of chicken lungs

Because the lung is one of major tissues where avian influenza virus (AIV) replicates in the chicken, we characterized whole-genome single-base DNA methylation profiles of chicken lung tissues from Fayoumi and Leghorn by the MethylC-seq. The same tissue samples were used in a previous study [25]).

**Fig. 1:**
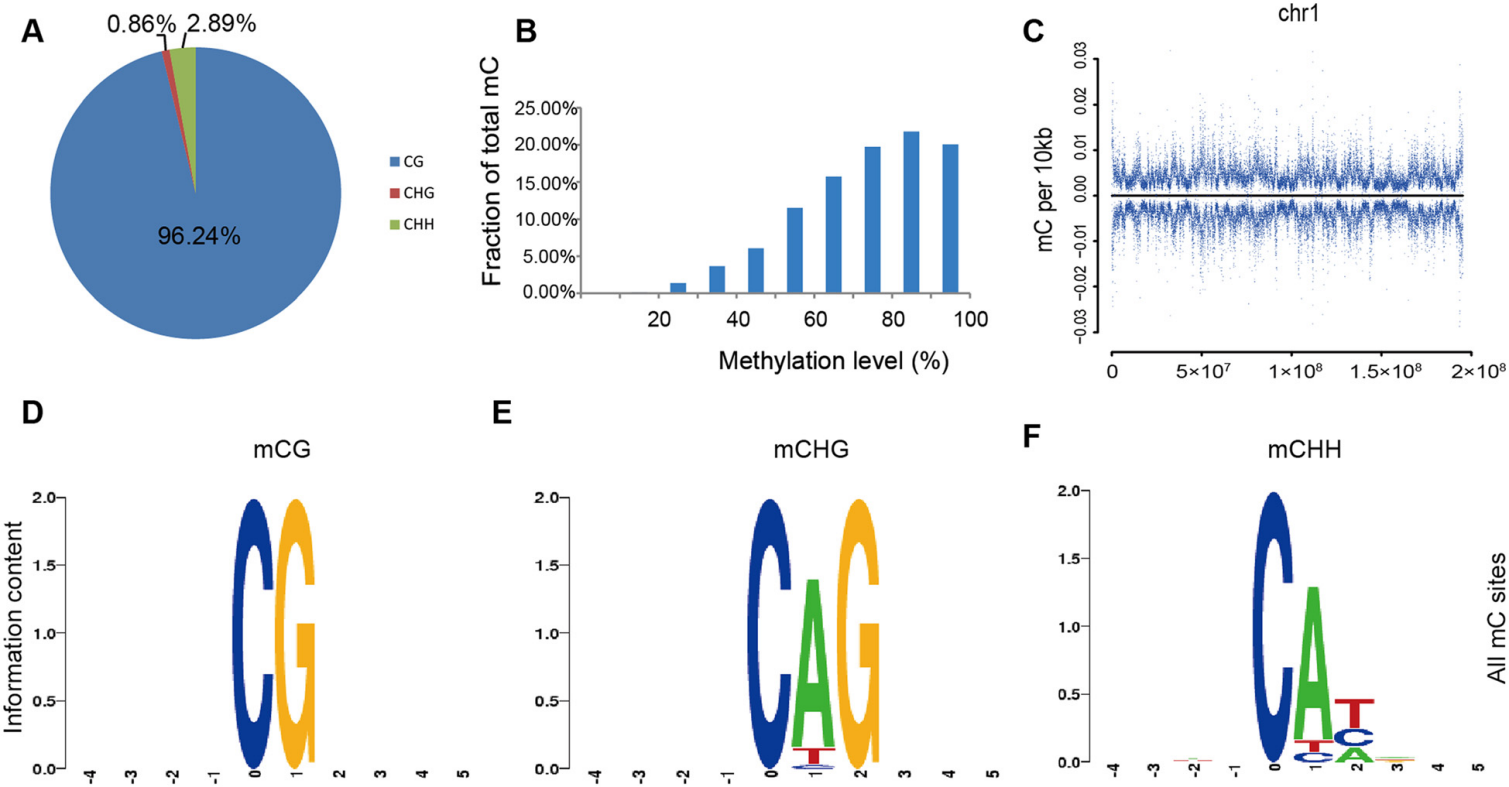
Global profile of the chicken DNA methylome. **a** The percentage of methylcytosines identified in chicken lungs. **b** Distribution of DNA methylation level in the CG context. The *y axis* means the fraction of all mCs that display each methylation level (*x axis*), where the methylation level is the mC/C ratio at each cytosine. **c** Blue dots indicate methylcytosine density in Leghorn lungs in 10-kb windows throughout the chromosome 1. The positive and negative value of *y axis* is the methylation density of the sense and antisense strand respectively. **d-f** Logo plots of the sequences proximal to sites of CG, CHG and CHH DNA methylation in each sequence context

A total of 148.82 gigabases (Gb) of sequence were generated from two biological replicates of each of two genetic lines. After filtering, 0.40 and 0.54 billion reads from Fayoumi and Leghorn lines, respectively, were uniquely mapped to the galGal4 reference sequence. Reads including more than three cytosines in non-CG contexts were considered as non-converted and removed. Then, we obtained 0.35 and 0.36 billion reads with an average read depth of 13.9× and 14.5 × per strand for Fayoumi and Leghorn, respectively (Table 1). The bisulfite conversion rates for all samples were 99.21 to 99.4 %. We used a binomial distribution to identify the methylcytosines, and 1 % of false discovery rate (FDR) was used to correct it. To validate the accuracy and repeatability of the methylation profiles, we compared the mCs identified independently in the two biological replicates, and found that nearly 90 % mCG sites were identical in the two individuals within each line, but only half of the mCs at non-CG sites were shared between lines (Additional file 1). To ensure the accuracy of our results, the intersection of methylcytosines in replicate 1 and replicate 2 were defined as mCs in each line. Finally, we detected approximately 11.3 and 11.7 million methylcytosines in Fayoumi and Leghorn, respectively, which represented about 3 % of all cytosines obtained by sequencing. More than half of the cytosines in CG contexts were methylated, whereas the methylation rates of the cytosines in CHG and CHH contexts (where H is A, C, or T) were only 0.11 and 0.12 %, respectively (Additional file 2). A total of 96.24 % of all methylcytosines occurred in the CG context, 0.86 % in the CHG context, and 2.89 % in the CHH context (Fig. 1a). To validate MethylC-seq results, we randomly examined methylation level of 165 mCGs, 61 mCHHs and 42 mCHGs using bisulphite PCR sequencing (BS-PCR). Of all the validated 165 mCGs, 96 % confirmed the sequencing results. However, none of the non-CG mCs were validated by the BS-PCR (Additional file 3).

**Table 1:** Sequencing results and read alignment

Samples	Library	Reads	Reads after filtered	Mapped reads	Uniq mapped reads	Post-processed	Bisulfite conversion rates	CpG coverage %
Fayoumi replicate 1	A	175421846	159533236	144001914	114988172	101197098	99.21 %	83.72 %
	B	175900866	151260322	118878023	79759740	68021164		
Fayoumi replicate 2	A	172418670	153201632	135605330	108313076	94229642	99.24 %	85.01 %
	B	170400106	151853856	127905703	96985346	85265402		
	All	694141488	615849046	526390970	400046334	348713306		
Leghorn replicate 1	A	200045434	178899768	160144382	126118192	87060156	99.33 %	86.92 %
	B	156119182	136244806	122471089	98190500	88847236		
Leghorn replicate 2	A	516069410	448675100	299761294	126087800	37391344	99.40 %	91.57 %
	B	293866348	268269302	239807905	191655228	151160202		
	All	1166100374	1032088976	822184670	542051720	364458938		

The chicken reference genome was downloaded from UCSC database (http://genome.ucsc.edu) Nov. 2011 (ICGSC Gallus_gallus-4.0/galGal4)

We defined the proportion of reads covering each methylcytosine relative to the total number of reads covering the sites as the methylation level given a specific cytosine. In the chicken lung, 62 % of mCG sites were 70–100 % methylated (Fig. 1b). We used chromosome 1 as an example to demonstrate the chromosome-wide DNA methylation density, and the CG methylation level revealed large variations across the chromosome 1, which was the same as other chromosomes (Fig. 1c, Additional file 4). The strand-specific mCGs were analyzed, where two strands showed a symmetrical methylation pattern. We also analyzed the genome sequence preference proximal to the sites of methylated CG and non-CG contexts. No sequence preference was found in the mCG-flanking regions or upstream of non-CG methylation; however, the base following a non-CG methylcytosine was almost always an adenine, while thymine was observed less often (Fig. 1d–f).

### Gene methylation profile

**Fig. 2:**
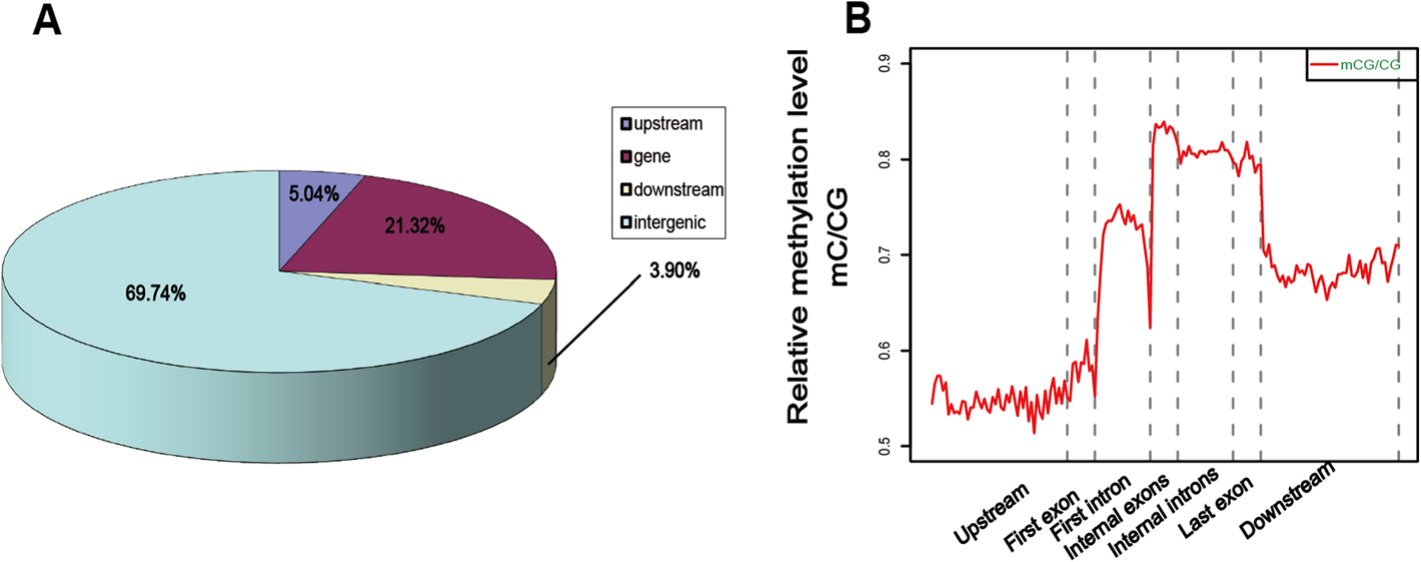
Distribution of methylated cytosines in different genome regions. **a** Proportion of methylated CpG islands in different genomic regions. **b** Relative methylation level in gene regions (Different areas were divided by dotted lines)

Methylation of CpG islands plays an important role in gene regulation during development, and it is altered in disease states [2, 32–34]. The CpG islands have been extensively studied since their identification more than 20 years ago [35]. In the present study, we characterized the CpG island methylation pattern in the chicken genome. To be characterized as a CpG island, a sequence must meet the following criteria: 1) (G + C) content above 55 %; 2) observed CpG/expected CpG of 65 % or greater; 3) more than 500 bp in length (http://methycancer.psych.ac.cn/CpG130.do) [36]; and 4) for methylated CpG island, the methylated CpG/ total CpG dinucleotide of 70 % or greater. By this definition, we identified 3767 methylated CpG islands. Among the 24,222 CpG islands, more than 80 % were un-methylated. Of all the methylated CpG islands, most (69.74 %) were in the intergenic regions. Only 5.04 % were in the gene upstream region and 3.90 % were in the downstream region, indicating that CpG islands at the 5' and 3' ends of genes were generally un-methylated. CpG islands in the gene-body regions (21.32 %) were more methylated than those in the 5’ and 3’ UTR regions (Fig. 2a).

To characterize methylation of chicken genes, we calculated the relative methylation levels (mC/CG) in the context of gene regions and of their upstream and downstream regions. We further divided the gene-body region into the first exon, first intron, internal exons, internal introns, and last exon. In general, the relative methylation level was higher in the gene-body regions than in the 5’ upstream and 3’ downstream regions. For the gene body, the relative methylation level was relatively low in the first exon and higher in the first intron. It reached the highest level in the internal exons and remained at a high level until the transcription termination site. Interestingly, there was always a sharp decrease of methylation across the exon-intron boundaries (dashed lines 2–5) (Fig. 2b).

**Fig. 3:**
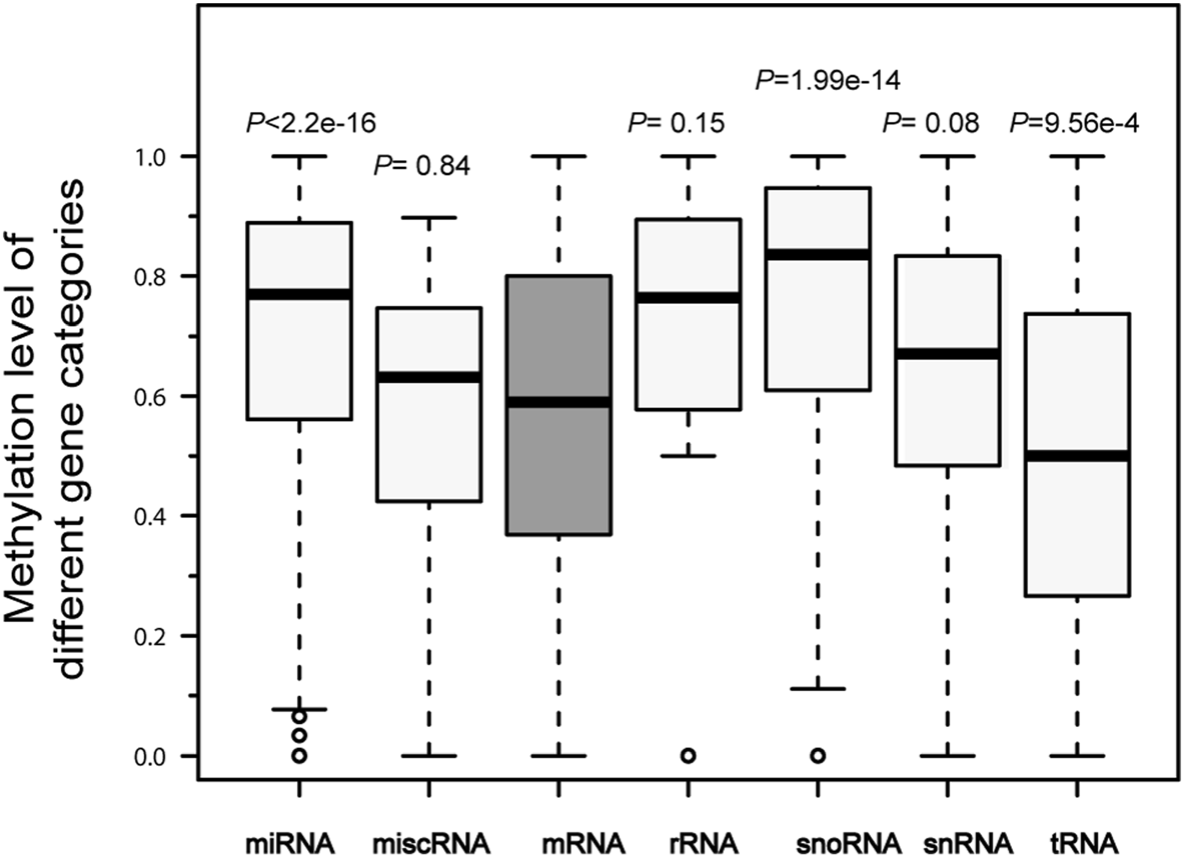
Promoter relative methylation level of different gene categories in the chicken genome. Box plots showed the methylation level of each gene category. Each category was compared with coding protein. miRNA (*P* <2.2E-16), misc_RNA (*P* = 0.8381), rRNA (*P* = 0.1472), snoRNA (*P* = 1.99E-14), snRNA (*P* = 0.08) and tRNA (*P* = 9.56E-4)

We next investigated the promoter methylation levels of different gene categories. A promoter region was defined as −1.5 kb to 0.5 kb relative to the TSS. Compared with protein-encoding genes, microRNA (miRNA) (*P* <2.2E^−16^) and small nucleolar (snoRNA) (*P* = 1.99E^−14^) were more highly methylated, and the tRNA (*P* = 9.56E^−4^) genes were less methylated, whereas miscellaneous RNA (misc_RNA) (*P* = 0.84), rRNA (*P* = 0.15) and snRNA (*P* = 0.08) did not show significant variance (*P* <0.05) (Fig. 3). Our results confirmed that miRNA genes were typically highly methylated [8, 37], suggesting that this phenomenon is highly conserved among species.

### DNA methylation and gene evolution in the chicken genome

**Fig. 4:**
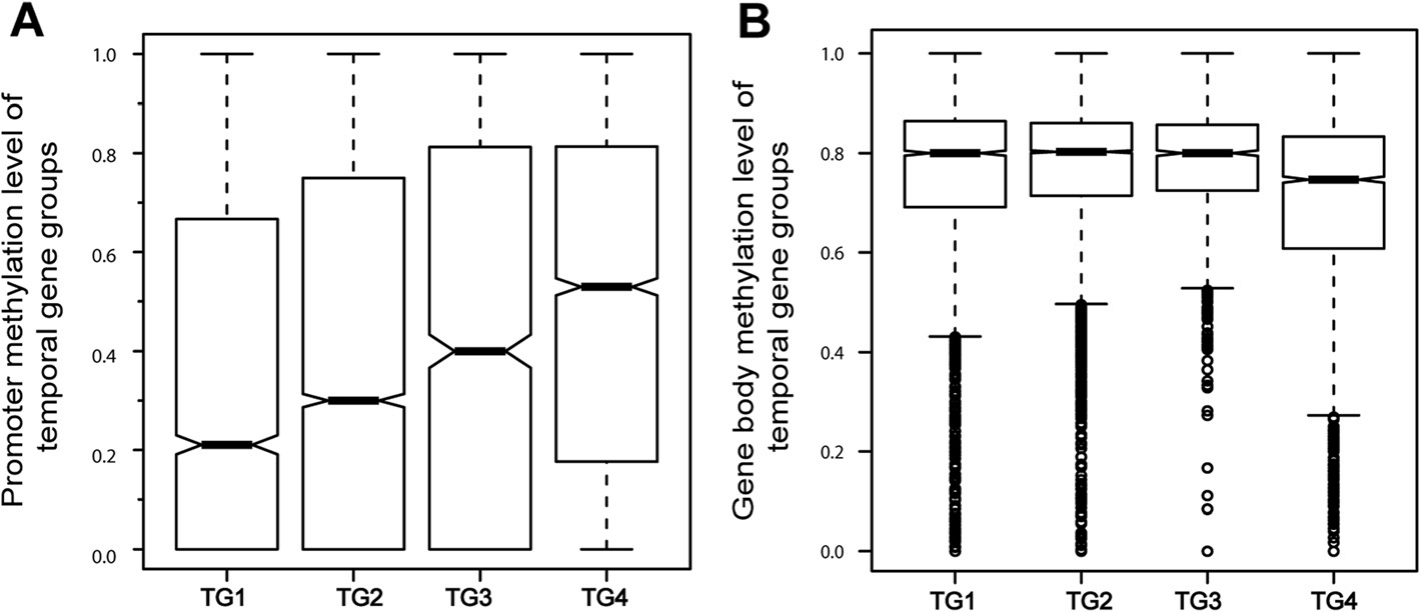
**a** Promoter relative methylation level of different temporal groups. The species used for each temporal group were: TG1 (African malaria mosquito, fruitfly, nematode, Schistosoma and yellow fever mosquito), TG2 (medaka, pufferfish, trout and zebrafish), TG3 (clawed frog and tropical frog), TG4 (all chicken genes not found in the above species). **b** Gene body relative methylation level of different temporal groups

To characterize evolutionary changes in gene methylation, we further classified chicken genes into four temporal groups based on a nucleotide sequence similarity search using BLAST against several clades in the evolutionary tree (hereafter referred to as TG, with TG1 being the oldest group; see the Methods section) [38]. We used the gene sequence to construct temporal groups. After that, we investigated the promoter and gene body methylation level of different temporal groups. Promoter methylation of the evolutionarily oldest genes had a significantly lower level (Student’s t-test, *P*-values were showed in Table 2), while the methylation level tended to increase with the evolution of genes (Fig. 4a). Gene body methylation level of different temporal groups was also performed and showed that gene body methylation of newly evolved genes was higher than early evolved groups (Fig. 4b).

**Table 2:** *P*-value between every two temporal gene groups

Promoter	TG2	TG3	TG4
TG1	4.40E-08	<2.2e-16	<2.2e-16
TG2		1.30E-08	<2.2e-16
TG3			6.21E-07
TG4			
Gene body	TG2	TG3	TG4
TG1	3.08E-09	2.26E-13	<2.2e-16
TG2		0.0003188	<2.2e-16
TG3			0.01255
TG4

The GO annotation analysis was conducted for hyper-methylated genes (methylation level ≥70 %) and hypo-methylated genes (methylation level ≤30 %) using WEGO (Web Gene Ontology Annotation Plot http://wego.genomics.org.cn/cgi-bin/wego/index.pl). Promoter and gene-body regions were separately analyzed. The gene list of each group is shown in Additional file 5. For the promoter region, most of the GO groups have more hypo-methylated genes, especially in cellular development, biological regulation and metabolic process. However, the molecular function with transducer and receptor activity, which is related to cellular responding to stimuli, establishment of localization, signaling and immune system processes, tend to have more hyper-methylated genes (Additional file 6). For the gene-body regions, almost all genes are hyper-methylated (Additional file 6), while hypo-methylated genes, which are rare, did not show any enrichment.

In addition, we also utilized DAVID functional annotation tool to perform the enrichment analysis (http://david.abcc.ncifcrf.gov/). The results were consistent with WEGO analysis, which showed that promoter hyper-methylated genes were significantly enriched in biological processes of stimuli, such as cognition, sensory perception and defense response. In the meanwhile, promoter hypo-methylated genes were clustered in transcription regulator activity (Additional file 7).

### DNA methylation distribution in the repeat elements and pseudogenes

**Fig. 5:**
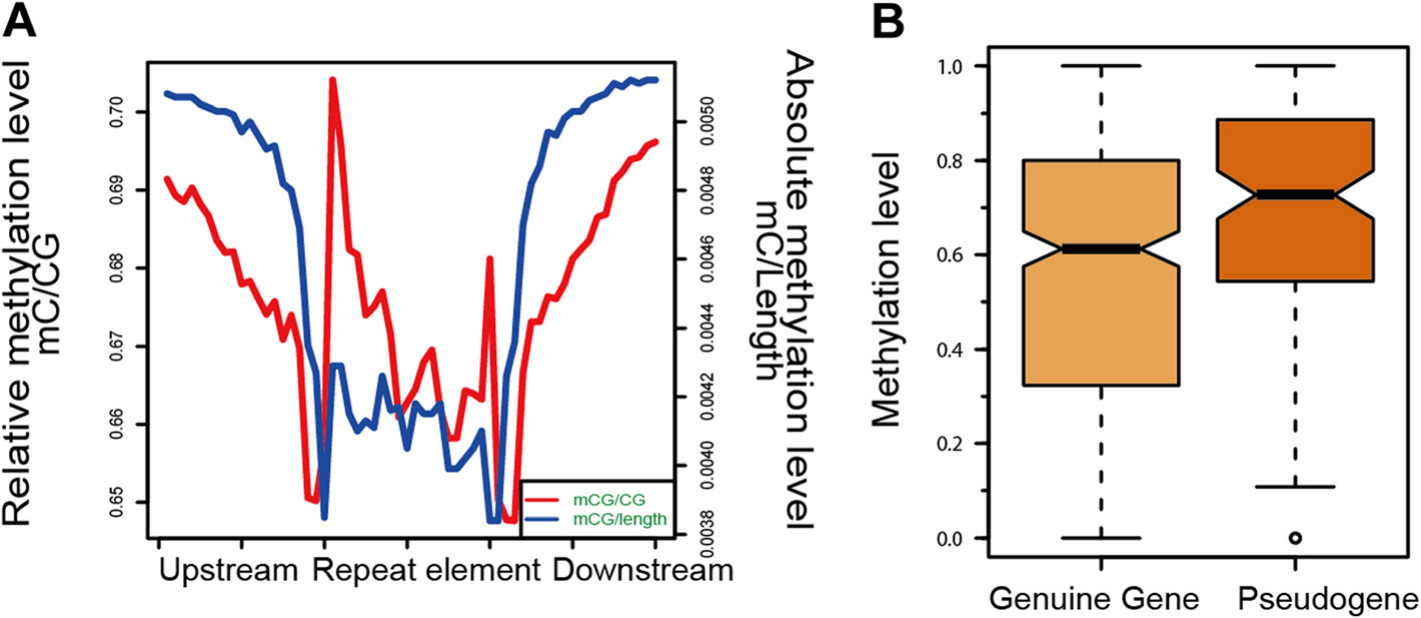
DNA methylation distribution in the repeat sequences and pseudogenes. **a** Absolute methylation level (*blue line*) and relative methylation level (*red line*) in repeat elements regions. **b** Promoter relative methylation level of pseudogenes and corresponding genuine genes

DNA methylation is essential for silencing transposable elements and other repetitive elements in eukaryotes. To investigate the effect on the regulation of repeat elements caused by DNA methylation in the chicken, we first confirmed the methylation level of repeat elements and its flanking regions. The absolute methylation level (mC/Length) in repetitive elements displayed a lower level than their flanking regions. With regard to the relative methylation level (mC/CG), the methylation density was lower in overall repetitive elements regions with the exception of a sharp increase in the boundary regions (Fig. 5a).

We then further compared the relative methylation level of different types of repeat elements (downloaded from UCSC database http://hgdownload.soe.ucsc.edu/downloads.html#chicken) and genome-wide randomly selected regions. For randomly selected regions, we excluded repeat sequences and genic regions (from 2 kb upstream of TSS to 2 kb downstream of TTS) to avoid the bias of sequence feature. In addition, the length and the number of repeat elements were also considered in the randomly selected regions. We observed that in chickens, all repeat elements had a lower relative methylation level than the randomly selected regions, except for mariner DNA repeats (Additional file 8).

We next compared the methylation level of pseudogenes with their corresponding genuine genes. The pseudogene information of chicken was downloaded from the pseudogene database (www.pseudogene.org) [39, 40], and the sequence information of pseudogenes and genuine genes were downloaded from the UCSC database. The pseudogenes’ promoter methylation levels were significantly higher than genuine genes (*P* =3.645e-05) (Fig. 5b).

### Correlation between DNA methylation and gene expression

**Fig. 6:**
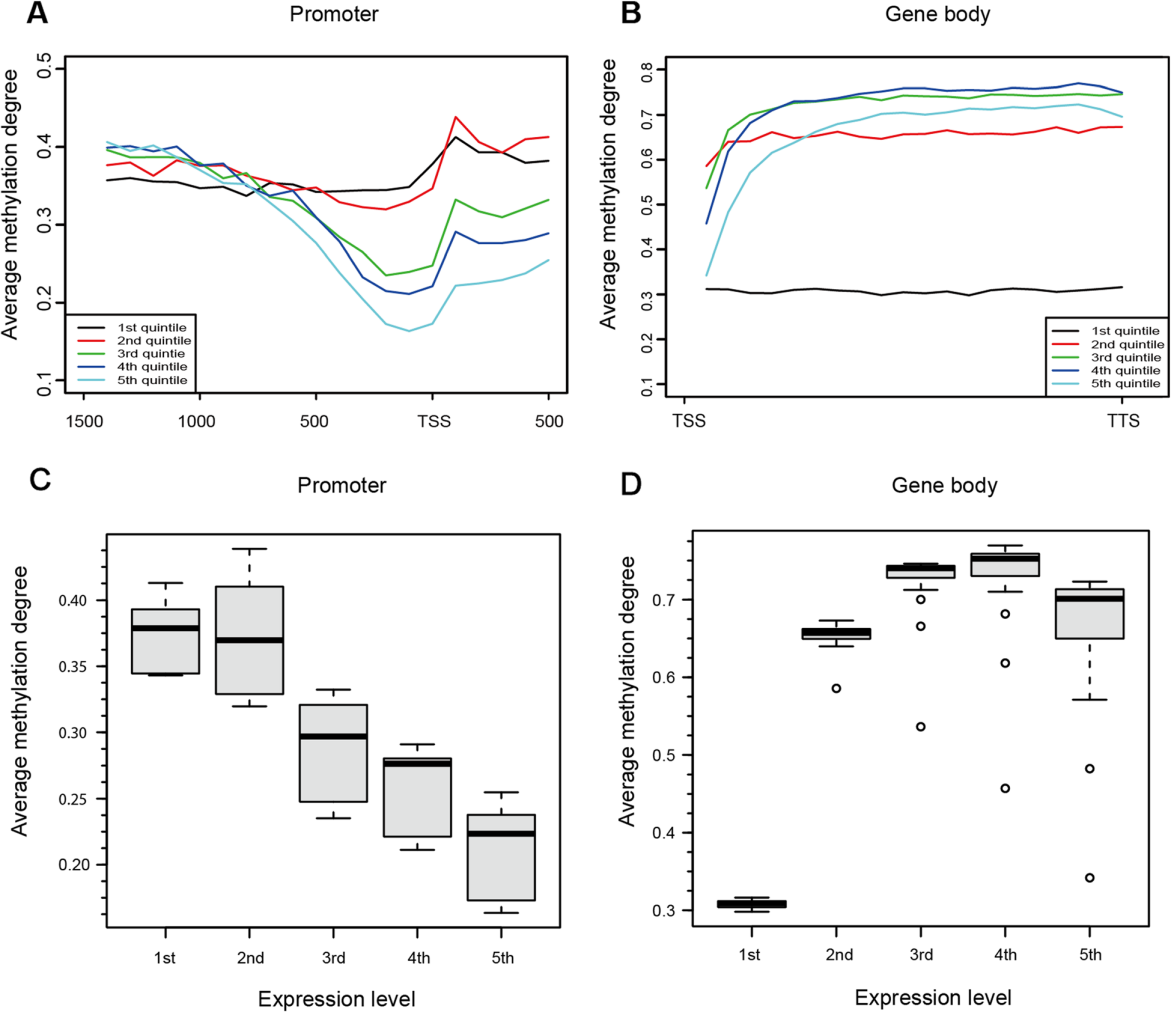
Relationship between DNA methylation and expression levels of genes in the chicken. **a-b** Average methylation degree across gene promoters and gene bodies. Genes were classified into five quintiles according to mRNA expression level: 1st quintile was the lowest and 5th was the highest. The promoter was defined as the region spanning from 1.5 kb upstream to 0.5 kb downstream of the transcript start site. **c-d** Box plots showed average methylation degree of promoters and gene bodies in each gene expression quintile

To analyze the correlation between average methylation degree (average methylation level of each CG sites) and expression of gene at mRNA level in these two genetic lines, RNA-seq profiles of chicken lungs from the same individuals that were used for MethylC-seq were generated. We divided the genes into five groups according to the mRNA expression level, from the bottom 20 % to the top 20 %, corresponding to the 1st to 5th quintiles. In general, the methylation degree across the five groups began to decrease from 1 kb upstream of the transcriptional start site (TSS) of the genes, and it increased after TSS (Figs. 6a, b). Box plotting showed that DNA methylation in the promoter region was negatively correlated with mRNA expression, especially at 500 bp around TSS (Figs. 6a, c). In contrast, the correlation between gene-body methylation and mRNA expression was more complex. In general, the expression level of the moderately expressed groups (2^nd^, 3^rd^ and 4^th^ quintiles) was positively correlated with the gene methylation. However, the methylation degree in the 5^th^ quintile (highest expressed group) was lower than the 3rd and 4^th^ quintiles (Fig. 6b, d). Methylation of the 1^st^ quintile was much lower than other groups.

### DNA methylation differences between two genetic lines

We characterized the methylation differences between the two genetic lines and explored how these methylation differences affected gene expression differences. To identify DNA methylation differences between the two genetic lines, we applied a sliding window method to identify the differentially methylated regions (DMRs) between Fayoumi and Leghorn chickens (5 % FDR). One-kilobase windows that contained at least four differential mCGs were identified, and adjacent windows were merged (see the Methods section). A total of 5652 DMRs were identified, among which 2400 DMRs were located within RefSeq genes (from 2 kb upstream to 2 kb downstream of gene). Based on these DMRs, we obtained 1532 DMR-associated genes (Additional file 9). Combined with RNA-seq results of the two lines [25], the DMRs were associated with 705 and 744 genes more highly expressed in Fayoumi and Leghorn, respectively (of 1532 DMR-associated genes, 83 showed no expression in both two chicken lines) (Additional file 10). And, 190 differentially methylated genes had more than two-fold differences in mRNA expression (Additional file 11).

We randomly selected *HCK* (a DMR-associated gene) to represent the DNA methylation and gene expression difference between the two chicken lines. MethylC-seq and BS-PCR validation results of ENSGALG00000006522 (*HCK*) were performed (Additional file 12), and differentially methylated region was highlighted by light gray (Additional file 12). We found that in the DMR, the methylation level of Fayoumi was higher than Leghorn and the BS-PCR validation result displayed an even more pronounced difference. In contrast, RNA-seq results showed that the expression of *HCK* was three times higher in Leghorn than in Fayoumi (Additional file 12).

The differentially methylated genes were further analyzed by gene ontology enrichment analysis using *DAVID*. Several immune-related GO terms including immunoglobulin domain, immune effector process and leukocyte mediated immunity were significantly enriched (Table 3). Of particular interest, several immune-related genes such as *TLR 4* and *PIK3CD* were both differentially methylated and differentially expressed between Fayoumi and Leghorn lines (Additional file 13).

**Table 3:** GO enrichment of DMR-associate genes

Gene ontology (GO) term	*P*-value for enrichment
Neuron differentiation	3.60E-04
Immunoglobulin domain	6.80E-04
Cell morphogenesis involved in differentiation	7.20E-04
Phosphate metabolic process	8.00E-04
Immune effector process	1.20E-03
Nucleoside binding	1.40E-03
Leukocyte mediated immunity	1.60E-03
Cellular component morphogenesis	2.10E-03
Enzyme activator activity	2.40E-03
Organelle lumen	3.00E-03

Promoter and gene body methylation level were calculated separately

**Fig. 7:**
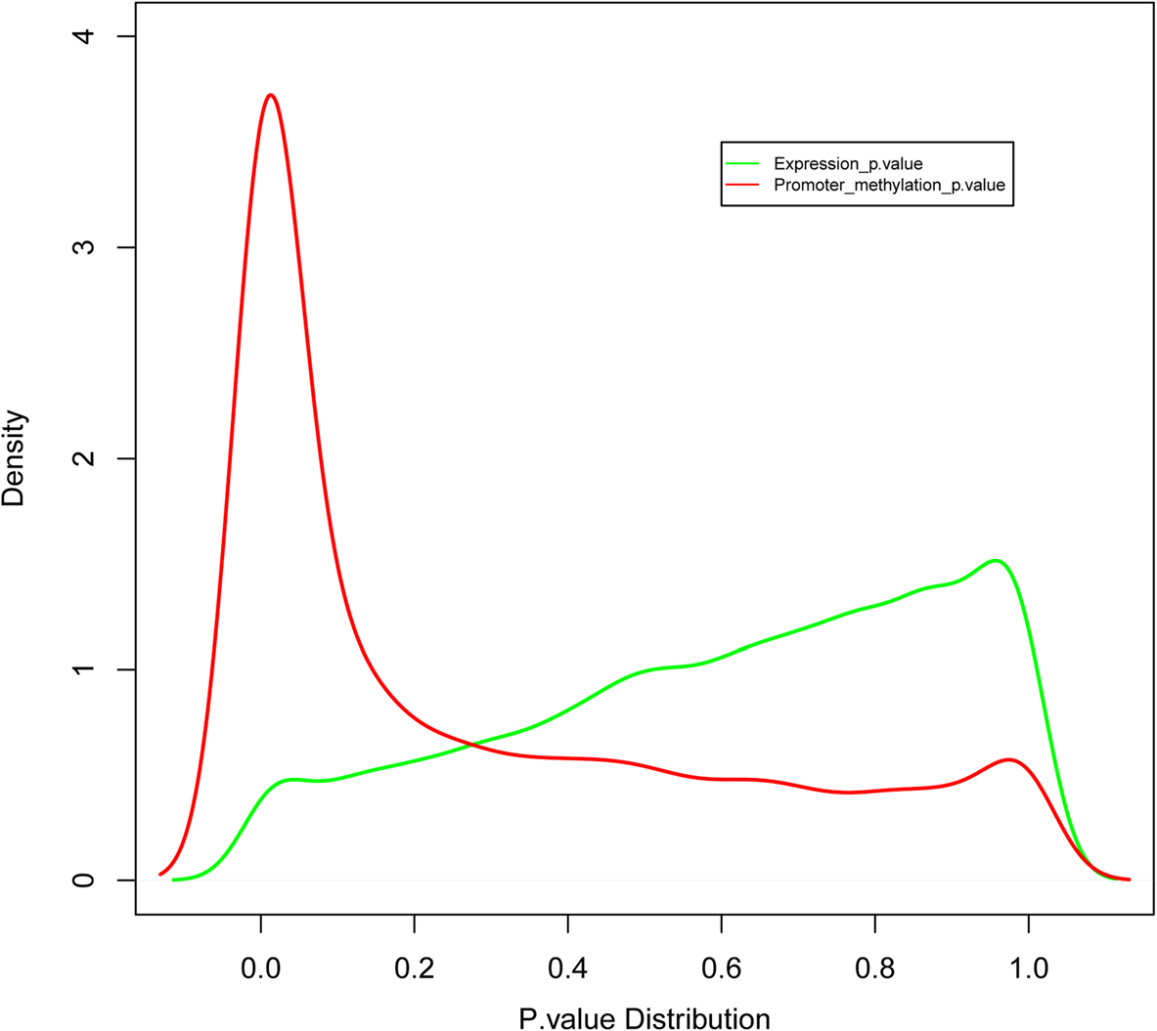
Genome wide gene expression and DNA methylation variation degree between Fayoumi and Leghorn. The transcription and DNA methylation *p*-value of each gene between two lines were calculated by *χ* ^2^ test

To evaluate the genome-wide gene expression and DNA methylation differences between Fayoumi and Leghorn, we obtained *p*-values of each gene between the two lines for transcription and DNA methylation by *χ*^2^ test and generated the distribution diagram. The results showed that the degree of DNA methylation variation between the two lines across the genome was greater than gene expression variation (Fig. 7). Furthermore, we analyzed the correlation between the whole genome promoter DNA methylation differences and gene expression differences but found no significant correlation (Additional file 14).

## Discussion

The stability of DNA methylation plays an important role in preventing tumorigenesis and disease progression [27, 28]. These results suggest that mediating gene regulation by DNA methylation may be associated with disease resistance. To improve our understanding of the relationship between DNA methylation and breed-specific disease resistance, we analyzed whole-genome single-base resolution DNA methylomes of Fayoumi and Leghorn chicken lungs. In this study, we report the single base DNA methylomes of chicken lungs and interrogate the potential role of DNA methylation on immune response.

Chicken methylomes have been previously characterized using MeDIP and Methyl-MAPS, which are techniques based on antibody binding affinity and restriction enzyme digestion [18–20]. Although both methods perform well for CpG-rich regions, they generate much lower resolution and coverage. The CpG coverage was only 32 % from the study in chickens using Methyl-MAPS [19]. In the current study, a total of 148.82 Gb sequencing data were generated, and the CpG coverage for each biological replicate ranges from 83.72 to 91.57 % (Table 1), which is comparable to both the human (94 %) and silkworm (92 %) methylomes [3, 8].

In the chicken, the general DNA methylation pattern is consistent with other species. For example, cytosine methylation occurs almost exclusively in the CG contexts; gene-body exhibits higher methylation than the 5’ and 3’ flanking regions [41–43]; promoter methylation negatively correlates with gene expression. These results suggest that the transcriptional regulatory role of DNA methylation is conserved among species [15, 44]. For the gene body regions, we found that internal exons show the highest methylation level, which is consistent with the study using Methyl-MAPS [19]. In addition, there was a great fluctuation of methylation across the exon-intron boundaries, which implies a potential link between DNA methylation and splicing [45]. In contrast with promoters, gene body methylation is positively correlated with gene expression except for the highest expressed group. It is in agreement with previous studies that both lowly and highly expressed genes have a low level of gene body methylation [5, 41, 42, 46]. A study in *Arabidopsis thaliana* suggests that this phenomenon was associated with formation of pre-initiation complexes and was directed by the siRNA pathway [47].

The results also revealed that the chicken DNA methylome has some different features than other species. Previous studies showed that one of primary functions of DNA methylation is host genome defense and targeting the endogenous transposable elements [48–51]. Further studies have proved that DNA methylation is a key regulator of transposon silencing in plants, some animals, and fungi, but not in invertebrates [42, 52]. In the chicken genome, the density of interspersed repeat elements is clearly lower than that in mammalian genomes [11]. Our study showed that the methylation level of repeat elements was lower than both their flanking regions and genome randomly selected regions. These results suggest that the phenomenon of DNA hyper-methylation in repeat elements existing in other vertebrates [42] may not exist in chickens. The chicken genome has relatively low transposable element activity [11]; therefore, we speculate that there may be no need to silence transposable elements through DNA methylation in the chicken.

In this study, we compared the promoter methylation levels in different gene categories. The results confirmed that the promoter regions of miRNAs in the chicken were highly methylated. Furthermore, analysis of the promoter methylation level of genuine genes and their corresponding pseudogenes showed that the methylation of pseudogenes was higher than that of genuine ones. Our results suggest that promoter hyper-methylation of miRNA and pseudogenes may suppress the expression of these genes, which is important to maintain the stability of chicken genome. In addition, we analyzed the methylation level changes related to gene evolution in chickens. The results suggest that the extent of gene methylation level was altered in different evolutionary stages. The newer gene group had higher methylation levels in the promoter regions, while the conserved ones had lower methylation levels. This result may arise from the phenomenon that ancient genes tend to be constitutively expressed [38, 53] and promoter methylation may be dispensable for that process. In contrast, the newest genes group is more likely to be tissue-specifically expressed [54] and, therefore, they may be more dependent on methylation regulation. Furthermore, we found that the gene body methylation level of the evolutionarily conserved genes was higher than that of the newest set of genes. This agrees with previous studies that demonstrated that gene body hyper-methylated genes were conserved and were functionally important [55, 56].

To expand our knowledge on the potential relationship between DNA methylation and disease resistance in chickens, the differentially methylated regions between two chicken lines that differ for disease resistance were identified. The many immune-related GO terms significantly enriched from DMR-associated genes between the two genetic lines suggest that DNA methylation may serve as one of the regulatory mechanisms that modulates immune response. Of particular note, some of the immune-related genes within DMRs also had significant mRNA expression differences between the two lines, including *PIK3CD* and *TLR4. PIK3CD* has been reported to play important roles in both innate and adaptive immunity; *PIK3CD* mutant mice showed decreased immune responses and impaired B/T cell development and function [57, 58]. *TLR4* (Toll-like receptor 4) is a member of the TLR family and plays a major role in pathogen recognition and activation of innate immunity [24]. Moreover, susceptible chickens have been reported to have an increased methylation level of the *TLR4* gene after *Salmonella* infection [59]. The results collectively suggest that DNA methylation may regulate host immune response via modulating expression of certain immune-related genes.

Of 1532 DMR-associated genes between the two lines, only 190 genes had significant expression differences (≥ two-fold changes). This finding is similar to previous studies in which only 6 % of differentially methylated genes had significant expression differences in *Arabidopsis thaliana* ecotypes, and 7.1 % in cultivated and wild rice [60, 61]. However, no significant correlation existed between genome wide-gene expression differences and promoter DNA methylation differences of the two lines, likely because DNA methylation is only one of many factors regulating transcription. Finally, a large number of genes showed significant differences in DNA methylation between the genetic lines, while a limited number of genes had significant differences in mRNA expression (Fig. 7). This suggests that DNA methylation may contribute more genetic differences between the two lines than transcription.

## Conclusion

This study provides the first report of single-base resolution methylation profiles in chicken tissues, which may serve as chicken reference epigenomes. We illustrate the regulatory role of DNA methylation in controlling gene expression and maintaining genome transcription stability. By profiling DNA methylomes of two unique highly inbred lines, our results also suggest the potential role of DNA methylation in regulating disease resistance in chickens.

## Methods

### Biological materials

Two genetically distinct, highly inbred chicken lines (Leghorn GB2 and Fayoumi M43) with an inbreeding coefficient of more than 99.99 % were used [26]. Two Leghorn and two Fayoumi birds (one male and one female chicken of each line) were euthanized at 3 weeks, and lungs were harvested. The animal experiment was performed according to the guidelines approved by the Institutional Animal Care and Use Committee, Texas A&M University.

### DNA preparation and MethylC-seq library generation

DNA from lung tissue was isolated by phenol-chloroform extraction. Five μg DNA was sonicated to produce 200–400 bp fragments, followed by end repair with a nucleotide triphosphate mix free of dCTP. Cytosine methylated adapters provided by Illumina (Illumina, San Diego, CA) were ligated to the sonicated DNA according to the manufacturer’s instructions for genomic DNA library construction. Adapter-ligated DNA with the length of 320–500 bp was isolated by 2 % agarose gel electrophoresis, and sodium bisulfite conversion was performed using the MethylEasy Xceed kit (Human Genetic Signatures, NSW, Australia) according to the manufacturer’s instructions. Then 300 ng of bisulfite-converted, adapter-ligated DNA molecules were enriched by 14 cycles of PCR with the following reaction composition: 2.5 U of uracil-insensitive Pfu TurboCx Hotstart DNA polymerase (Stratagene), 5 μL of 10× Pfu Turbo reaction buffer, 25 μM dNTPs, 1 μL of Primer 1.1, and 1 μL of Primer 2.1 (50 μL final). The thermocycling parameters were as follows: 98 °C for 2 min, followed by 4 cycles of 98 °C for 15 s, 60 °C for 30 s, and 72 °C for 1 min, ending with incubation at 72 °C for 10 min. The reaction products were purified using the MinElute PCR purification kit (Qiagen, Valencia, CA) and then separated by 2 % agarose gel electrophoresis and purified by the MinElute gel purification kit (Qiagen, Valencia, CA).

### High-throughput sequencing

The DNA libraries were sequenced using the Illumina Genome Analyzer II (GA II) according to the manufacturer’s protocols. The MethylC-seq libraries were subjected to 80 or 81 cycles to yield longer sequences that are more amenable for unambiguous mapping to the galGal4 genome reference sequence. The chicken reference genome was downloaded from UCSC database (http://genome.ucsc.edu) Nov. 2011 (ICGSC Gallus_gallus-4.0/galGal4). Two independent libraries from each biological replicate (line) were sequenced so that parallel analysis was performed on each biological replicate to insure the accuracy of sequencing result.

### Processing and alignment of MethylC-seq

Read sequences produced by the Illumina pipeline in the FastQ format were first pre-processed. Reads containing more than three cytosines in a non-CG context were considered unconverted sequences and removed [3]. Then, the reference sequence was prepared and simultaneously converted twice as follows: (1) cytosines were replaced with thymines and (2) guanines were replaced with adenines. Following pre-processing, the reads were sequentially aligned to two computationally converted reference sequences using the BWA. All results from the alignment of a read to both the Watson and Crick converted genome sequences were combined, and if more than one alignment position existed for a read, it was categorized as ambiguously aligned and disregarded. We removed reads that shared the same 5’ alignment position within each library, referred to as “clonal” reads, leaving the first read. Reads mapped to the wrong strands were discarded (T-rich reads mapped to Crick-strand Cs converted to Ts or to Watson-strand Gs converted to ‘A’s, A-rich reads mapped to Watson-strand Cs converted to Ts or to Crick-strand Gs converted to ‘A’s) [62]. Subsequently, the reads from all libraries of a particular sample were combined. All unambiguous, or “unique”, read alignments were then subjected to post-processing.

### Identification of methylated cytosines

To identify methylcytosines, we used binomial distribution and then 1 % false discovery rate (FDR) was used to correct the *P*-value. We kept the number of false positives methylcytosine calls below 1 % of the total number of methylcytosines identified. The probability *p* in the binomial distribution B (n, *p*) was estimated from the number of cytosine bases sequenced in reference cytosine positions in the unmethylated mitochondria genome (Error rate: non-conversion plus sequencing error frequency) [63, 64]. For each reference cytosine, the number (n) is the read depth, and the cytosine is noted as methylated if the number of sequenced cytosines (m) follows the following formula as below [62]:

$$ {C}_n^m{p}^m\left(1-p\right)\mathrm{n}-\mathrm{m}<0.01\mathrm{m}/\left(\mathrm{n}\hbox{-} \mathrm{m}\right) $$

For each biological replicate, the reads from two technical replicates (A and B) were pooled to provide greater coverage for the identification of the methylcytosines. The methylcytosines presented in this study represent the consensus between two biological replicates.

### Identification of differentially methylated regions (DMRs)

We first used Fisher’s Exact Test to find the differentially methylated cytosines between Fayoumi and Leghorn with 5 % FDR correction and at least two-fold difference in methylation level (mCG/CG reads). Then, a sliding window method was used to search for differentially methylated regions between Fayoumi and Leghorn line. A 1 kb window containing at least four differential mCGs was considered as an initial seed, and moved 100 bp per iteration in both the 5’ and 3’ directions. When a 1 kb window containing at least four differential mCGs was identified, the region was extended in 100 bp increments until a 1 kb increment was reached that contained less than four differential mCGs. After the extension in both directions, regions that contained at least 5 differential mCGs and were at least 1 kb long were identified as DMRs [3]. These DMRs were joined together by removing the overlapping region on the same chromosome.

### cDNA Library Preparation and Sequencing by RNA-Seq

Two lung RNA samples from the same genetic line were pooled to generate a total of two pooled RNA samples. Total RNA (7 μg) was subjected to two rounds of hybridization to oligo (dT) beads (Invitrogen, Carlsbad, CA) to enrich mRNA. Ribosomal RNA contamination was evaluated by RNA pico chip using a BioAnalyzer (Agilent, Santa Clara, CA). The resulting mRNA was then used to prepare cDNA libraries using the RNA sequencing sample preparation kit (Illumina, San Diego, CA). The two libraries were sequenced by Illumina Genome Analyzer II, which generated two datasets.

### Data filtering, mapping reads and transcriptome analysis

The sequences generated were initially subjected to a filtering process. Any reads that contained numerous interspersed Ns in their sequences, or had relatively short reads (<17 bp), were removed for subsequent analysis. Sequence reads obtained after quality control with filtering were analyzed using CLC Genomics Workbench 4 (CLC bio, Cambridge, MD). After mapping, the unique gene reads for all of the 17,108 annotated chicken genes in the database from the two libraries were combined, and analyzed using the DESeq R package [65]. Differentially expressed genes between two genetic lines were identified at combined fold change >2. The RNA-seq number showed in Additional file 10 is normalized unique gene reads. Genes with low absolute reads (below 10) had been removed. Statistics related to over representation of functional categories were performed using DAVID [66–68].

### Chicken gene temporal groups construction

All chicken genes were divided into four temporal groups based on the nucleotide sequence similarity according to the clades in the evolutionary tree (including insect, fish, amphibians, birds and mammals) and searched using BLAST with an E-value threshold set to e^−20^. Specifically, if a chicken gene had sequence homology with fruitfly, mosquito, and nematode or schistosoma species over the threshold, it was classified into the oldest temporal group. If a second gene had a homolog in the pufferfish, medaka, trout or zebrafish species but not in the first clade was placed in the second temporal group. The species used for each temporal group are: TG1 (African malaria mosquito, fruitfly, nematode, Schistosoma and yellow fever mosquito), TG2 (medaka, pufferfish, trout and zebrafish), TG3 (clawed frog and tropical frog), TG4 (all chicken genes not found in the above species).

### BS-PCR validation

One microgram of genomic DNA from each biological replicate was bisulfite-converted by the EZ DNA Methylation-Gold™ Kit. Primers were designed to amplify target regions of the bisulfite-converted DNA for validation of the MethylC-Seq results. Then we randomly selected 10–15 TA clones for each PCR product and sequenced by Sanger sequencing. All the primers were listed in Additional file 15.

### Availability of supporting data

The sequencing data from this study have been submitted to the NCBI Gene Expression Omnibus (http://www.ncbi.nlm.nih.gov/geo) under accession no. GSE56975.

## Additional files

Additional file 1:
**Correlation analysis of the two biological replicates.** Overlap of mCs in CG, CHG and CHH contexts between the two biological replicates. mCs were classified as unique to biological replicate 1 (yellow), unique to replicate 2 (red) or shared by both replicates (orange). The number of overlapped mCs in each category was listed, as well as the percentage of mCs unique within each biological replicate. (TIFF 362 kb) Additional file 2:
**Number of cytosines and methylcytosines detected in the chicken genome.** (DOCX 16 kb) Additional file 3:
**Bisulfite sequencing validation results on CG sites.** (XLS 72 kb) Additional file 4:
**Methylcytosine density in a 10-kb window of all chromosomes in chickens.** (PDF 8163 kb) Additional file 5:
**List of promoter and gene body hypo- and hyper-methylated genes.** (XLSX 63 kb) Additional file 6:
**GO enrichment of promoter and gene body hyper- or hypo-methylated genes.** (**A-B**) GO enrichment of hyper-methylated genes (methylation level≥70 %) and hypo-methylation genes (methylation level≤30 %) in promoter (A) and gene body (B). Annotations are grouped by biological process, cellular component and molecular function based on the Gene Ontology database (http://www.geneontology.org/). Gene numbers are listed for each category. (TIFF 657 kb) Additional file 7:
**DAVID functional enrichment of promoter and gene body hypo- and hyper-methylated genes.** (XLSX 80 kb) Additional file 8:
**The relative methylation level of different repeat types and genome random selected regions.** (DOC 43 kb) Additional file 9:
**DMR-associated genes between Fayoumi and Leghorn lines.** (XLSX 205 kb) Additional file 10:
**Expression levels of DMR-associated genes from RNA-seq data.** (XLSX 161 kb) Additional file 11:
**DMR-associated genes that show more than two-fold expression differences between Fayoumi and Leghorn.** (XLSX 33 kb) Additional file 12:
**Methylation distribution of a specific DMR-associated gene.** (**A**) Methylation distribution of DMR-associated gene *HCK* (ENSGALG00000006522) was performed by UCSC genome browser custom tracks. Fayoumi and Leghorn lines were demonstrated separately. The bars indicate the methylation level of each mCG sites. Differentially methylated region was highlighted in light gray. (**B**) Bisulphite-PCR validation of some different methylated CG sites within the light gray region. Orange part represents the methylation percentage of each site. (**C**) RNA-seq result of *HCK* in Fayoumi and Leghorn birds. (TIFF 1128 kb) Additional file 13:
**Methylation distribution and expression level of DMR-associated gene** ***TLR4 and PIK3CD*** **in Fayoumi and Leghorn.** (**A**) Methylation distribution of the differentially methylated regions in *TLR4* and *PIK3CD* by the UCSC genome browser custom track. Fayoumi and Leghorn lines were demonstrated separately. The bars indicate the methylation level of each mCG sites. Differentially methylated regions were highlighted in light gray. (**B**) RNA-seq result of the DMR-associated gene between Fayoumi and Leghorn lines. (PDF 1737 kb) Additional file 14:
**Correlation analysis between DNA methylation differences and gene expression differences.** X axis represented expression fold change and Y axis represented promoter methylation fold change of each gene. (PDF 95 kb) Additional file 15:
**PCR primers used for Bisulfite sequencing validation.** (XLSX 12 kb)

## Abbreviations

DMR: Differentially methylated region
TSS: Transcriptional start site
TTS: Transcriptional terminal site
TG: Temporal group
