# Supplementary figures and images for: Genome-wide DNA methylome variation in two genetically distinct chicken lines using MethylC-seq
Source: BMC Genomics. 2015 Oct 23;16:851. doi: 10.1186/s12864-015-2098-8 (PMC4619007; doi:10.1186/s12864-015-2098-8)

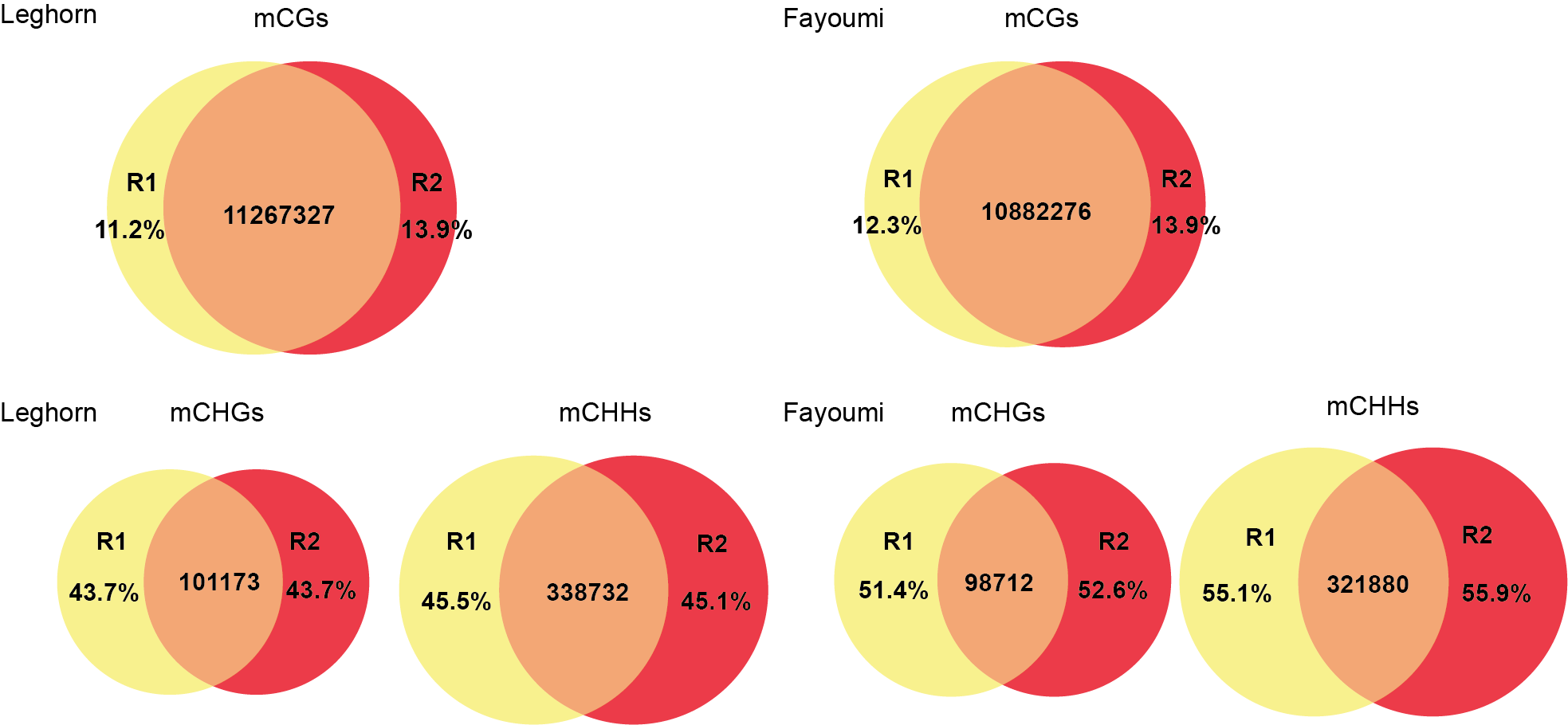

Supplement: Additional file 1: — Correlation analysis of the two biological replicates. Overlap of mCs in CG, CHG and CHH contexts between the two biological replicates. mCs were classified as unique to biological replicate 1 (yellow), unique to replicate 2 (red) or shared by both replicates (orange). The number of overlapped mCs in each category was listed, as well as the percentage of mCs unique within each biological replicate. (TIFF 362 kb) [file 12864_2015_2098_MOESM1_ESM.tiff]

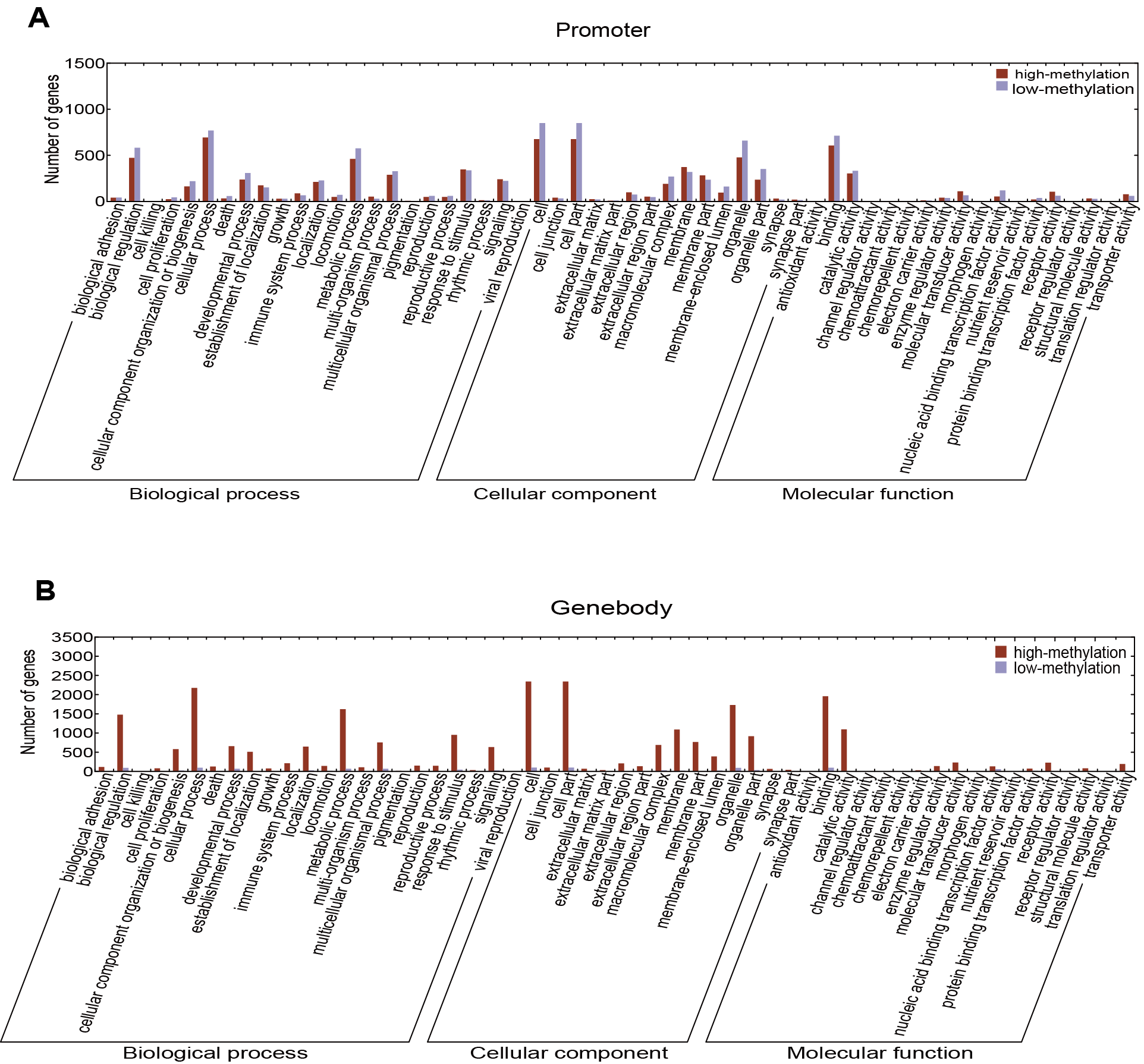

Supplement: Additional file 6: — GO enrichment of promoter and gene body hyper- or hypo-methylated genes. (A-B) GO enrichment of hyper-methylated genes (methylation level≥70 %) and hypo-methylation genes (methylation level≤30 %) in promoter (A) and gene body (B). Annotations are grouped by biological process, cellular component and molecular function based on the Gene Ontology database (http://www.geneontology.org/). Gene numbers are listed for each category. (TIFF 657 kb) [file 12864_2015_2098_MOESM6_ESM.tiff]

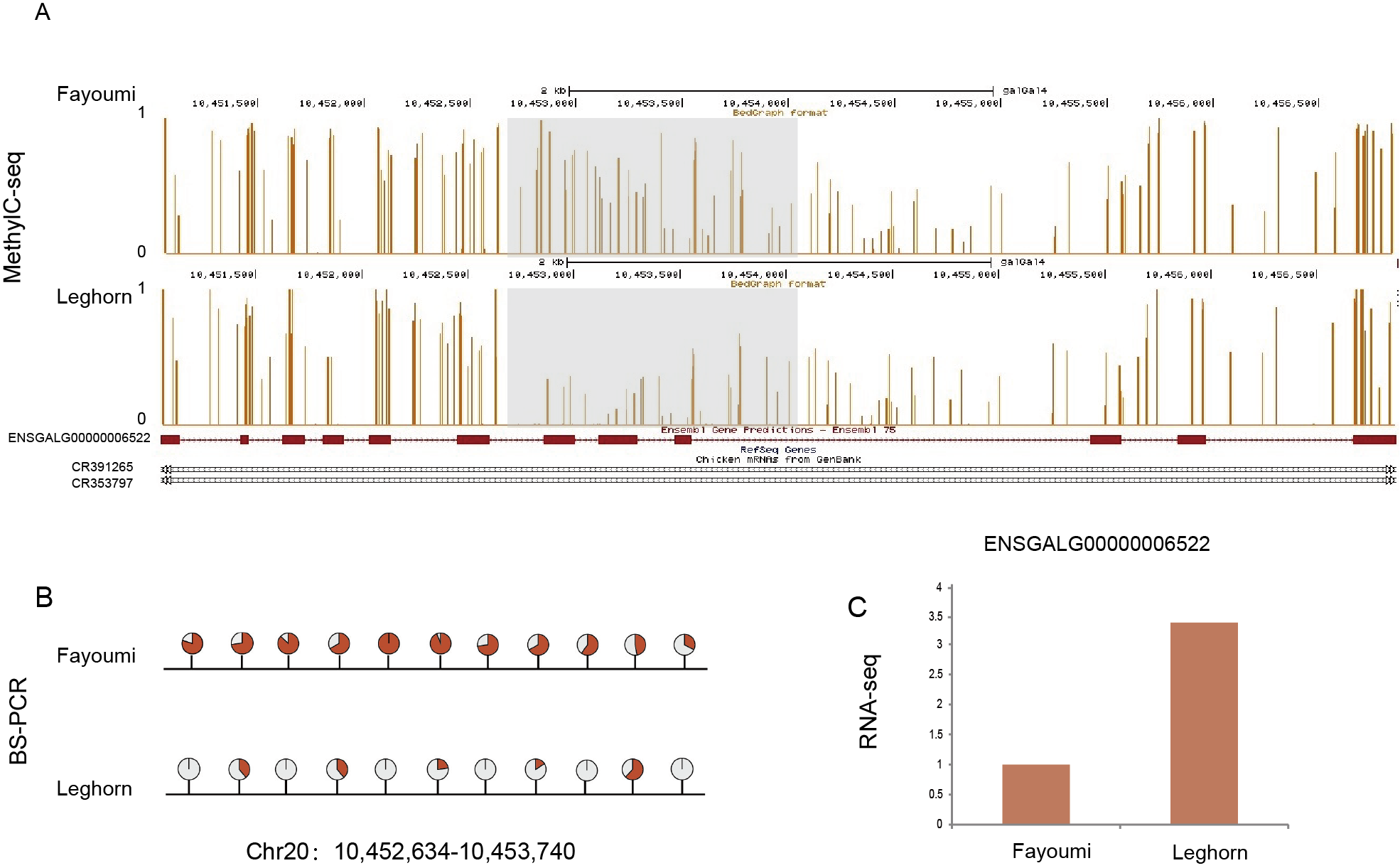

Supplement: Additional file 12: — Methylation distribution of a specific DMR-associated gene. (A) Methylation distribution of DMR-associated gene HCK (ENSGALG00000006522) was performed by UCSC genome browser custom tracks. Fayoumi and Leghorn lines were demonstrated separately. The bars indicate the methylation level of each mCG sites. Differentially methylated region was highlighted in light gray. (B) Bisulphite-PCR validation of some different methylated CG sites within the light gray region. Orange part represents the methylation percentage of each site. (C) RNA-seq result of HCK in Fayoumi and Leghorn birds. (TIFF 1128 kb) [file 12864_2015_2098_MOESM12_ESM.tiff]

**A** ENSGALG00000007001- TLR 4

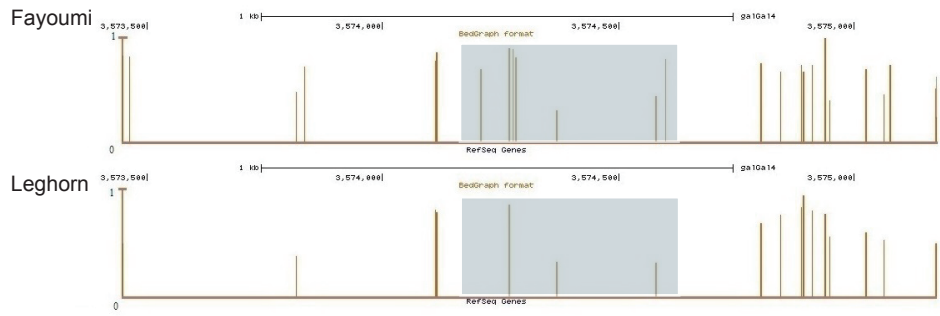

**B** ENSGALG00000002583- PIK3CD

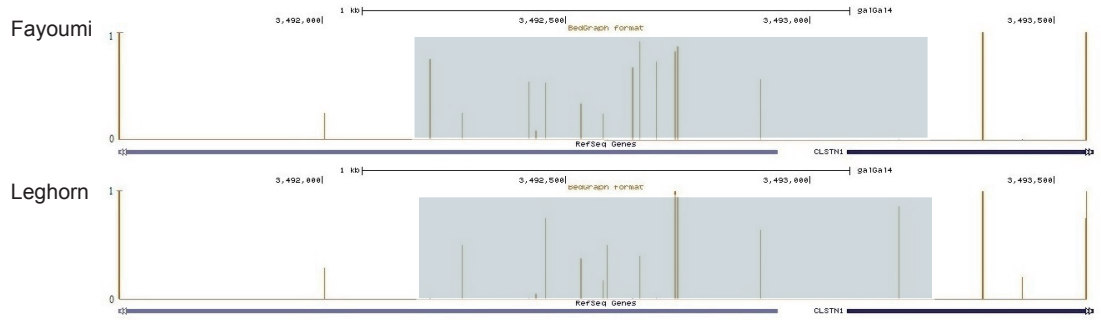

**C**

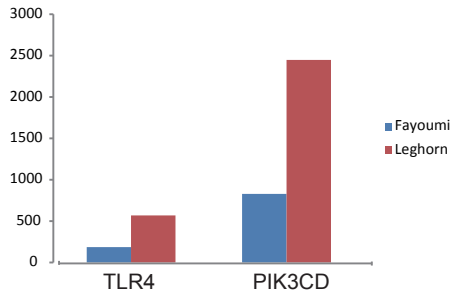

Supplement: Additional file 13: — Methylation distribution and expression level of DMR-associated gene TLR4 and PIK3CD in Fayoumi and Leghorn. (A) Methylation distribution of the differentially methylated regions in TLR4 and PIK3CD by the UCSC genome browser custom track. Fayoumi and Leghorn lines were demonstrated separately. The bars indicate the methylation level of each mCG sites. Differentially methylated regions were highlighted in light gray. (B) RNA-seq result of the DMR-associated gene between Fayoumi and Leghorn lines. (PDF 1737 kb) [file 12864_2015_2098_MOESM13_ESM.pdf]

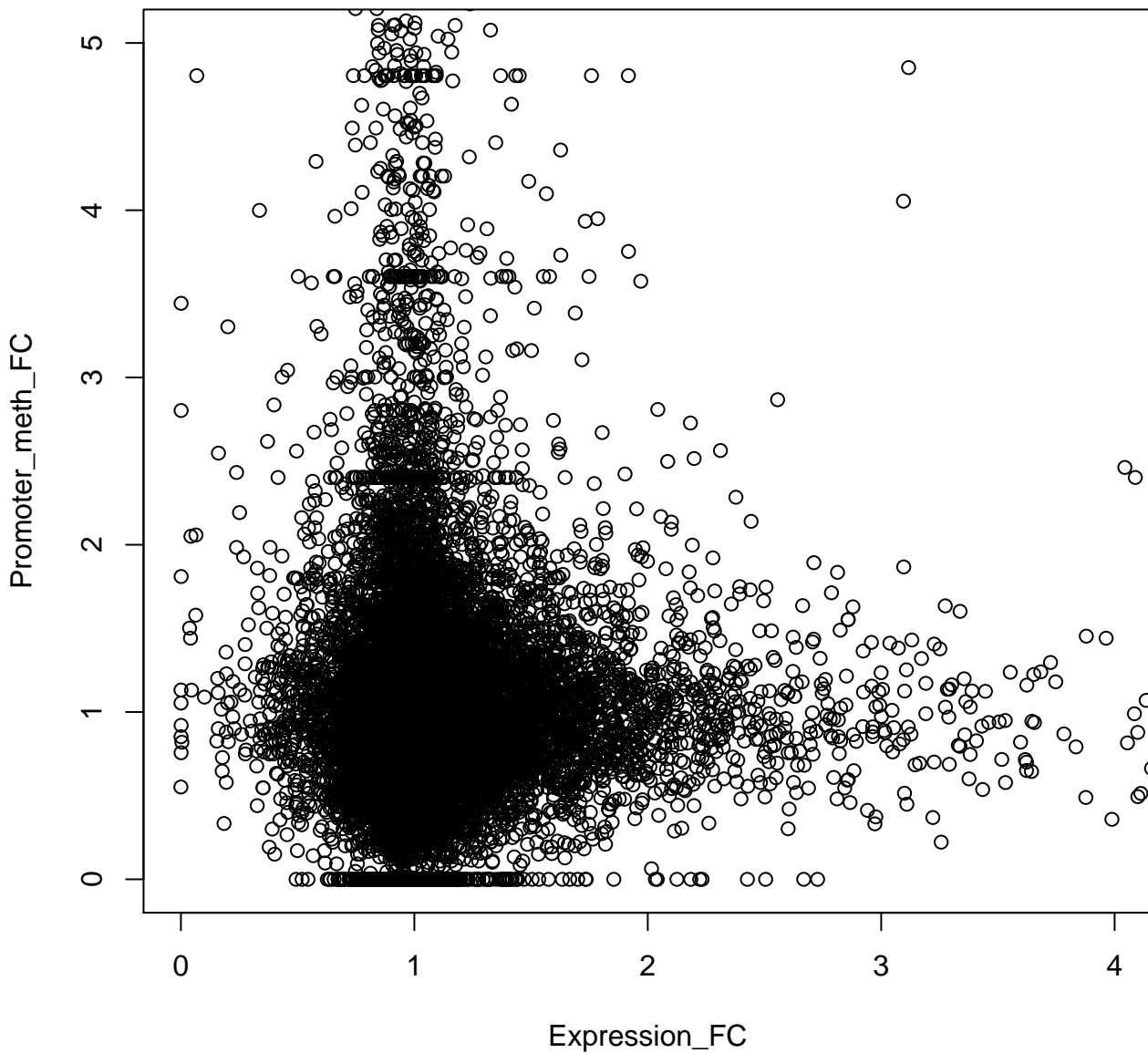

Supplement: Additional file 14: — Correlation analysis between DNA methylation differences and gene expression differences. X axis represented expression fold change and Y axis represented promoter methylation fold change of each gene. (PDF 95 kb) [file 12864_2015_2098_MOESM14_ESM.pdf]
