# Supplementary material for: Genome-wide DNA methylome variation in two genetically distinct chicken lines using MethylC-seq
Source: BMC Genomics. 2015 Oct 23;16:851. doi: 10.1186/s12864-015-2098-8 (PMC4619007; doi:10.1186/s12864-015-2098-8)
Supplement: Additional file 2: — Number of cytosines and methylcytosines detected in the chicken genome. (DOCX 16 kb) [file 12864_2015_2098_MOESM2_ESM.docx]

**Number of cytosines and methylcytosins detected in chicken genome**

| **sample** | **CG** | **mCG** | **mCG%** | **CHG** | **mCHG** | **mCHG%** | **CHH** | **mCHH** | **mCHH%** | **mC%** |
| --- | --- | --- | --- | --- | --- | --- | --- | --- | --- | --- |
| **Fayoumi** | 19541073 | 10882276 | 55.69% | 93045790 | 98712 | 0.11% | 259383661 | 321880 | 0.12% | **3.03%** |
| **Leghorn** | 19845782 | 11267327 | 56.77% | 95707569 | 101173 | 0.11% | 272488557 | 338732 | 0.12% | **3.02%** |
