# Supplementary material for: Genome-wide DNA methylome variation in two genetically distinct chicken lines using MethylC-seq
Source: BMC Genomics. 2015 Oct 23;16:851. doi: 10.1186/s12864-015-2098-8 (PMC4619007; doi:10.1186/s12864-015-2098-8)
Supplement: Additional file 8: — The relative methylation level of different repeat types and genome random selected regions. (DOC 43 kb) [file 12864_2015_2098_MOESM8_ESM.doc]

**The relative methylation level of different repeat types and genome random selected regions**

| Sample |  | FC（%） | LC（%） |
| --- | --- | --- | --- |
| Repeat type | CpG/100 bp |
| DNA/Charlie | 0.883 | 57.64 | 60.47 |
| DNA/mariner | 0.844 | 71.95 | 75.49 |
| DNA/piggybac | 0.836 | 44.17 | 47.04 |
| Satellite | 1.837 | 20.48 | 21.61 |
| LTR/ERV1 | 2.018 | 43.18 | 44.49 |
| LTR/ERVK | 3.019 | 58.24 | 59.14 |
| LTR/ERVL | 1.024 | 44.02 | 47.04 |
| LINE | 0.608 | 35.35 | 41.43 |
| LINE/CR1 | 0.827 | 56.26 | 57.33 |
| Low complexity | 1.933 | 1.06 | 1.05 |
| Simple repeat | 0.841 | 2.42 | 2.52 |
| SINE/Deu | 0.908 | 40.51 | 44.26 |
| SINE/MIR | 0.835 | 34.71 | 37.64 |
| SINE/tRNA | 0.793 | 43.24 | 46.91 |
| snRNA | 1.844 | 33.36 | 36.77 |
| rRNA | 2.562 | 42.02 | 40.73 |
| tRNA | 5.874 | 26.20 | 27.59 |
| Genome randomly selected regions | 2.94 | 64.99 | 68.68 |
