# Supplementary material for: Genome-wide DNA methylome variation in two genetically distinct chicken lines using MethylC-seq
Source: BMC Genomics. 2015 Oct 23;16:851. doi: 10.1186/s12864-015-2098-8 (PMC4619007; doi:10.1186/s12864-015-2098-8)
Supplement: Additional file 4: — Methylcytosine density in a 10-kb window of all chromosomes in chickens. (PDF 8163 kb) [file 12864_2015_2098_MOESM4_ESM.pdf]

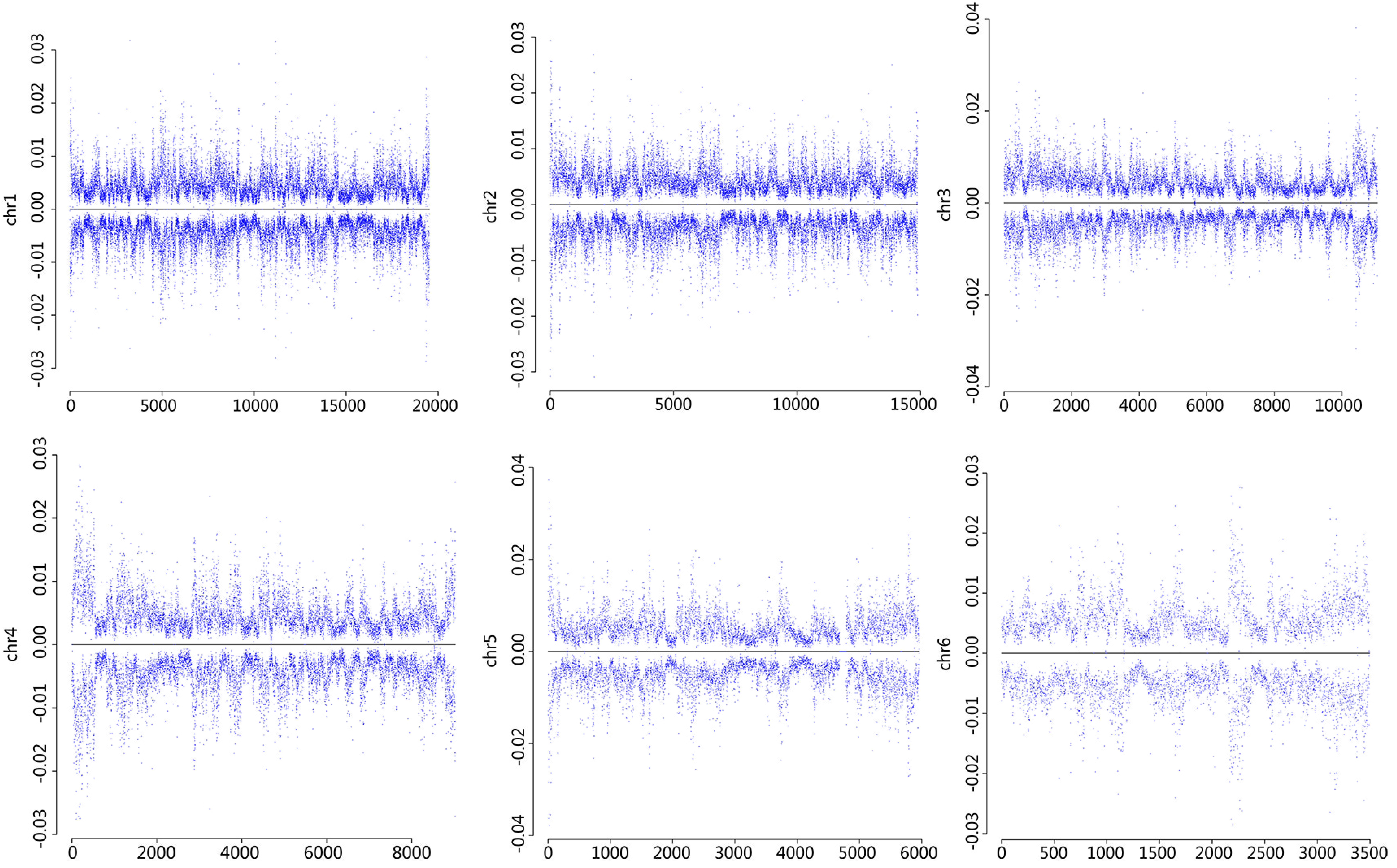
chr1
0.03
0.02
0.01
0.00
-0.01
-0.02
-0.03
0
5000
10000
15000
20000
chr2
15000
chr3
0.04
-0.04
2000
4000
6000
8000
chr4
chr5
1000
3000
5000
chr6
500
1500
2500
3500

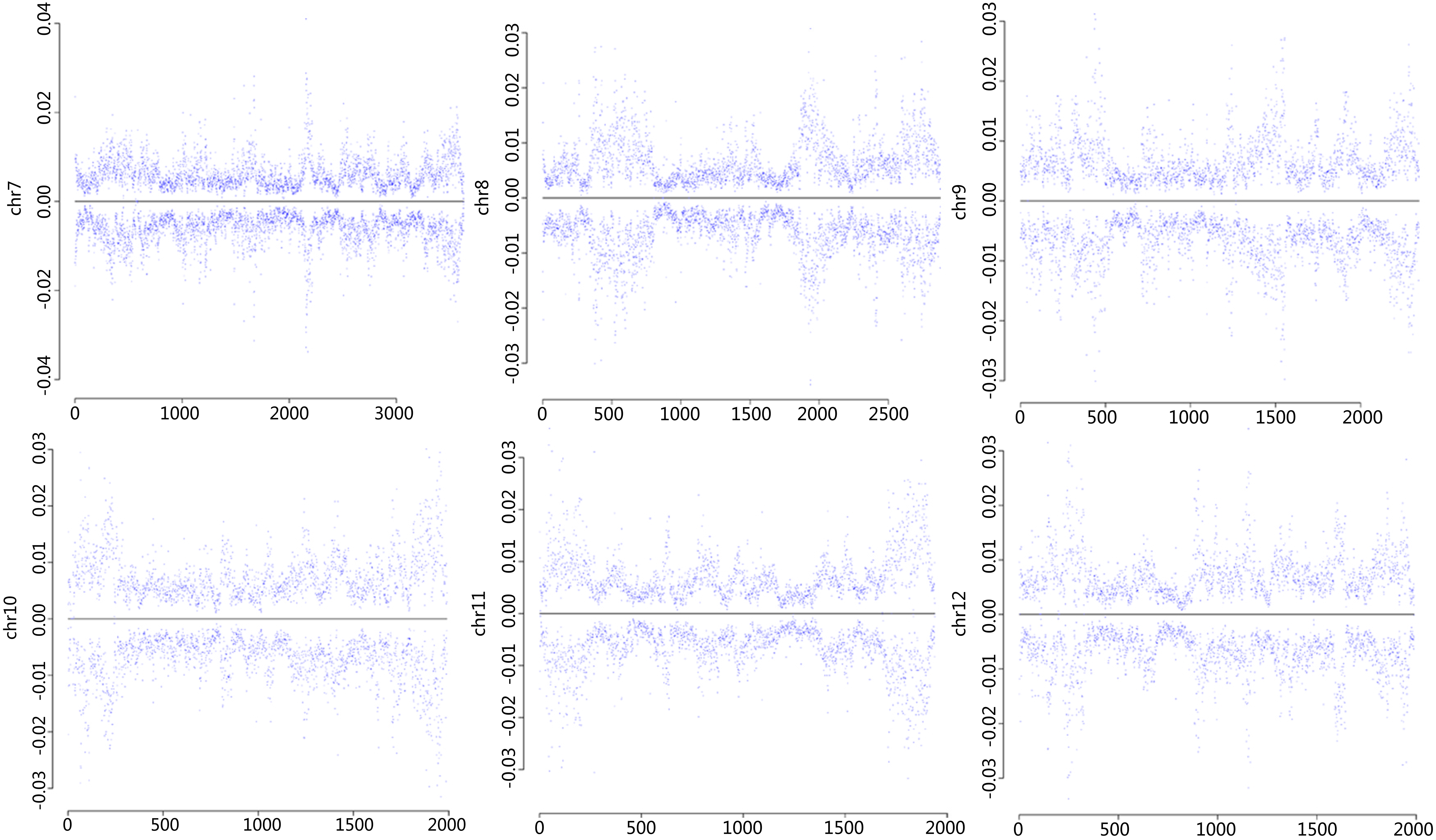

chr7
0.04
0.02
0.00
-0.02
-0.04
0
1000
2000
3000
chr8
0.03
0.02
0.01
0.00
-0.01
-0.02
-0.03
0
500
1000
1500
2000
2500
chr9
0.03
0.02
0.01
0.00
-0.01
-0.02
-0.03
0
500
1000
1500
2000
chr10
0.03
0.02
0.01
0.00
-0.01
-0.02
-0.03
0
500
1000
1500
2000
chr11
0.03
0.02
0.01
0.00
-0.01
-0.02
-0.03
0
500
1000
1500
2000
chr12
0.03
0.02
0.01
0.00
-0.01
-0.02
-0.03
0
500
1000
1500
2000

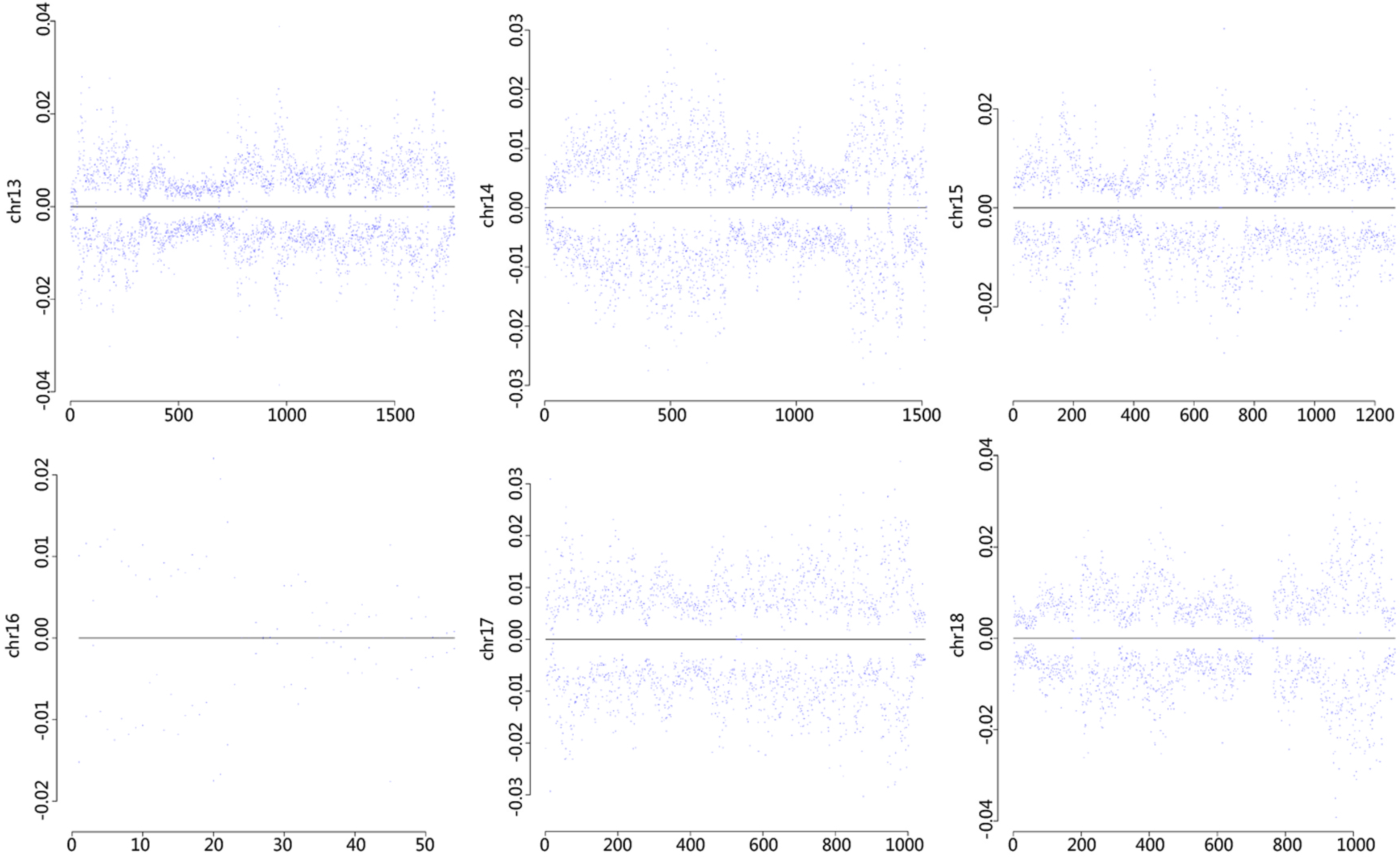
chr13
0.04
0.02
0.00
-0.02
-0.04
0
500
1000
1500
chr14
0.03
0.02
0.01
0.00
-0.01
-0.02
-0.03
0
500
1000
1500
chr15
0.02
0.00
-0.02
0
200
400
600
800
1000
1200
chr16
0.02
0.01
0.00
-0.01
-0.02
0
10
20
30
40
50
chr17
0.03
0.02
0.01
0.00
-0.01
-0.02
-0.03
0
200
400
600
800
1000
chr18
0.04
0.02
0.00
-0.02
-0.04
0
200
400
600
800
1000

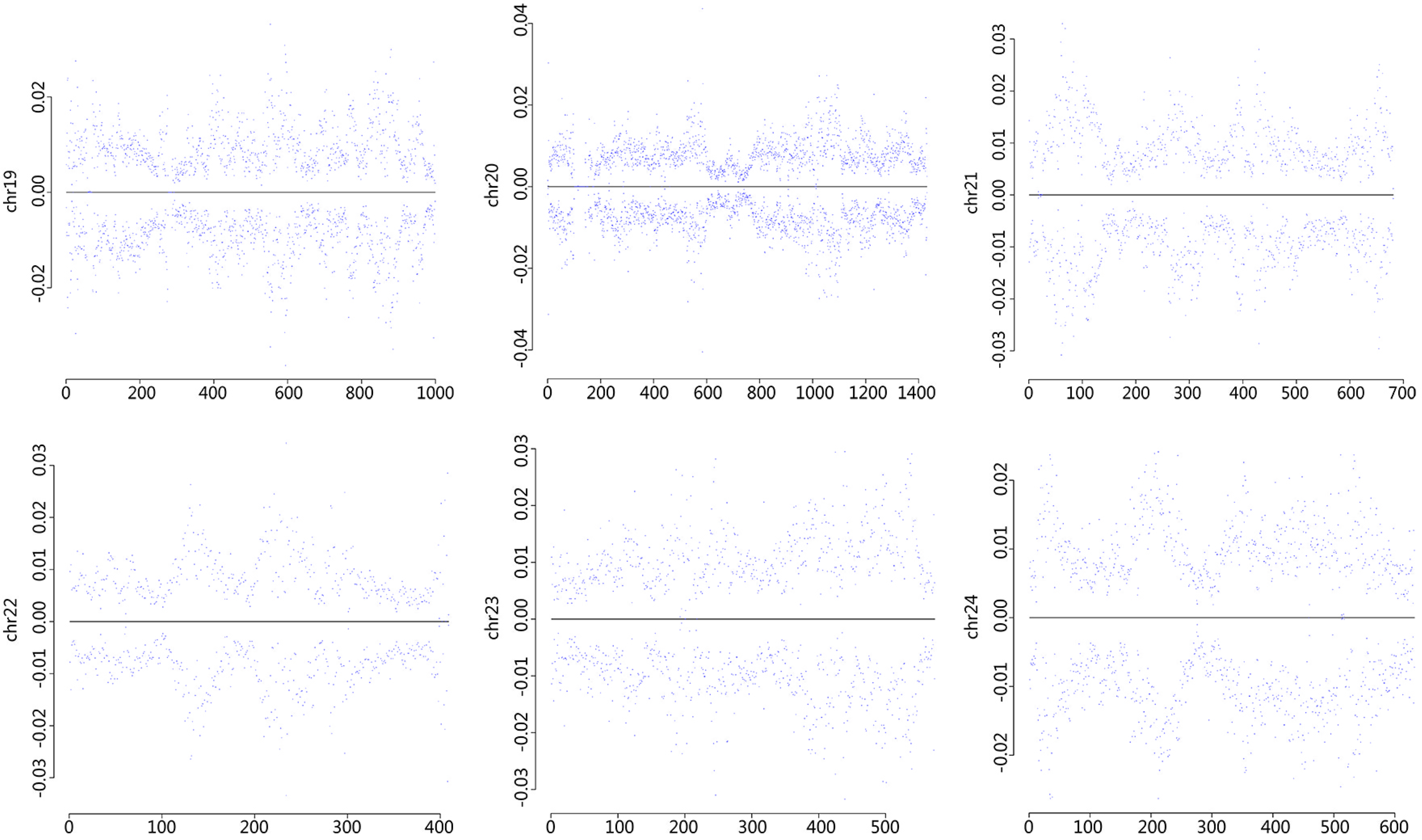
chr19
chr20
chr21
chr22
chr23
chr24

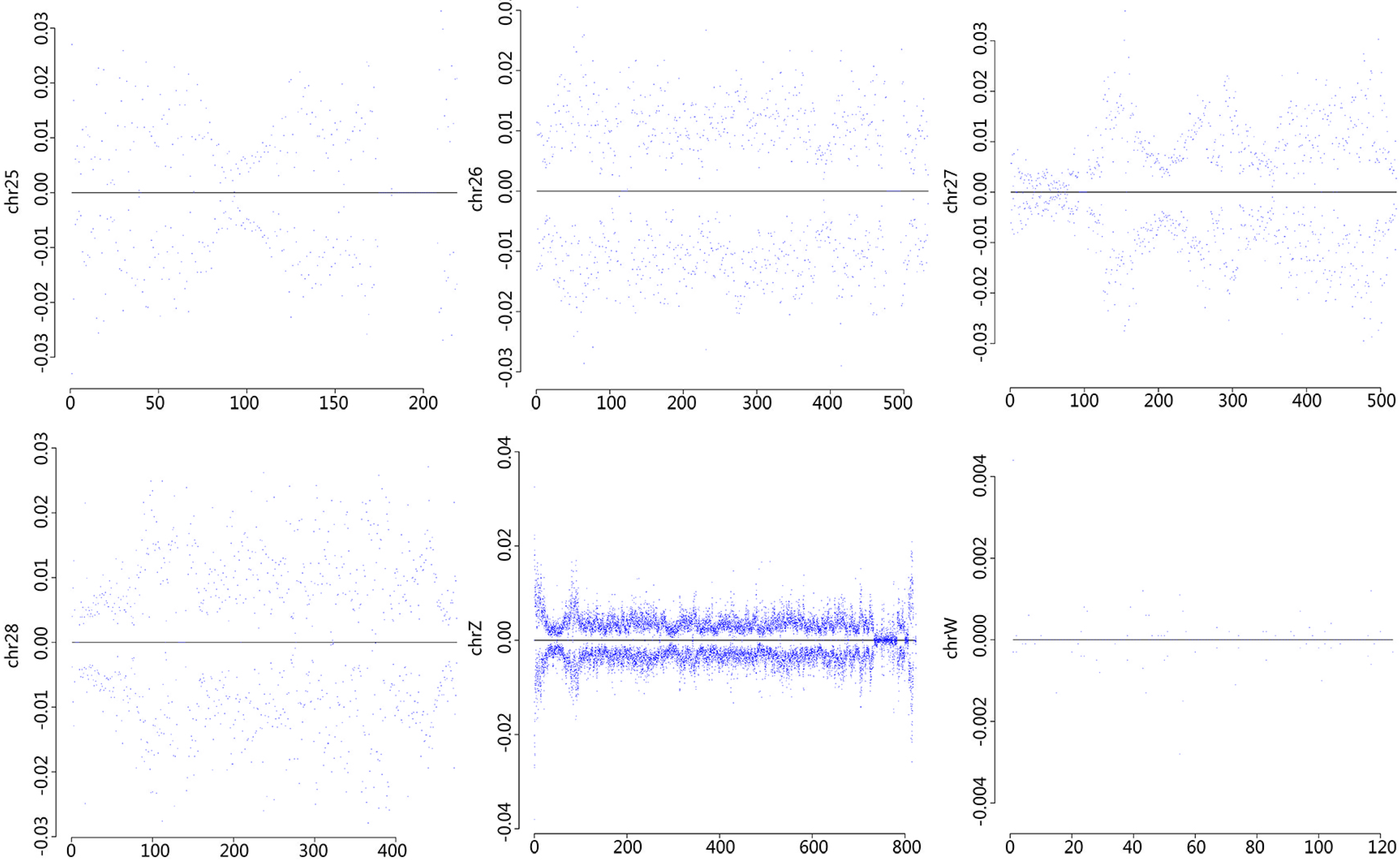
chr25
chr26
chr27
chr28
chrZ
chrW
